# Suhuang Antitussive Capsules-Ameliorative Effects on LPS-Induced Sputum Obstruction in Mice Through Promoting HGF Secretion

**DOI:** 10.3389/fphar.2019.01422

**Published:** 2019-12-19

**Authors:** Xiyang Tong, Rongyao Liang, Yuning Jia, Weiwei Qin, Chao Guo, Xingdong Wu, Zhen Wang, Dong Chen, Ninghua Tan

**Affiliations:** ^1^State Key Laboratory of Natural Medicines, Department of TCMs Pharmaceuticals, School of Traditional Chinese Pharmacy, China Pharmaceutical University, Nanjing, China; ^2^Yangtze River Pharmaceutical Group Beijing Haiyan Pharmaceutical Co., Ltd., Beijing, China; ^3^Beijing University of Chemical Technology, Beijing, China

**Keywords:** Suhuang antitussive capsule, MUC5AC, hepatocyte growth factor, sputum obstruction, airway inflammation

## Abstract

Sputum obstruction is one of common cough complications, which is tightly associated with airway inflammation. Suhuang antitussive capsule (SH Capsule), a classic traditional Chinese medicine prescription, has been used for the treatment of post-cold cough and cough variant asthma in the long clinical application. This study aims to investigate the effects and underlying mechanisms of SH Capsule on LPS-induced sputum obstruction in mice. The results showed that SH Capsule effectively promoted the tracheal phenol red output and mucociliary clearance. SH Capsule also alleviated airway inflammation-mediated mucin 5AC (MUC5AC) level through EGFR-ERK signaling. A further *in vivo* analysis showed that HGF inhibitor SU11274 abrogated the effects of SH Capsule on MUC5AC, well demonstrating that HGF was required for the beneficial effects of SH Capsule on expectoration *in vivo*. Moreover, SH Capsule promoted HGF secretion in a colon-dependent manner, which reached lung tissues *via* blood circulation. Collectively, this study provided new pharmacological data for clinical use of SH Capsule, and proposed a novel mechanism by which SH Capsule was pharmacologically promising for treating sputum obstruction.

## Introduction

Respiratory disease is a widespread healthy problem over the world, whose main lesions are in the trachea, bronchus, lungs and chest, leading to restricted activity and a worsening quality of life (Naghavi et al., 2017). People with respiratory diseases are commonly accompanied by airway inflammatory diseases such as chronic obstructive pulmonary disease (COPD), bronchial asthma, lung cancer, and diffuse lung diseases (Hastie et al., 2017). These may be tightly associated with mucin (MUC) hypersecretion in sputum. Sputum is located in the airway luminal surface, under normal circumstances, goblet cells in the bronchial mucosa secrete a small amount of mucus to keep the respiratory mucosa moist and clear impurities, such as ash layer and infectious agents, which plays critical roles in maintaining the normal physiological state of the respiratory tract. However, in pathological conditions, massive exudates such as fibrin and mucus, inhaled dust and some tissue necrosis are mixed to form a sputum obstruction. Sputum obstruction is one of the major manifestations in chronic inflammatory-associated respiratory diseases. During the progression of COPD and severe acute asthma attacks, massive mucus secretion in the airways can lead to airway obstruction, airflow limitation, and even mucus plug formation (Zhou et al., 2013; Bone et al., 2017). Commonly, inflammatory infiltration and hypertrophy of airway epithelial cells can induce airway wall thickening and trachea narrowing. The previous studies have proposed that inflammation-mediated airway lesion weakens mucosal movement and increases adhesion protein secretion, which subsequently increases sputum viscosity (Whitehead et al., 2017). In this forward cycle of inflammation occurrence conditions, the defect in sputum obstruction is also responsible for severe cough (Demarche et al., 2016). Nevertheless, there are few safe and effective therapeutics currently available for expectorant-associated respiratory diseases. Thus, it is imperative to find out a satisfactory therapeutic drug for this disease.

The increase of sputum viscosity is the main reason why sputum is difficult to cough up, and the high expression of mucin protein in sputum will directly lead to the increase of sputum viscosity, indicating that the over-expression of mucin protein is crucial for the sputum obstruction. There are four major types of gel-forming mucins-mediated sputum in the airway, such as MUC2, MUC5AC, MUC5B and MUC19 (Williams et al., 2006). Among them, MUC5AC has been the most extensively investigated one, linking to mucus hypersecretion in the pulmonary tracts as well as COPD, asthma, lung cancer and bronchiectasis (Wang et al., 2012; Song et al., 2016). MUC5AC is present in the surface epithelial cell layer, and mostly expressed in the airway secretion protein, resulting in a extremely close relationship with mucus hypersecretory diseases (Alessandra et al., 2017). Additionally, Zhang et al. have reported that ambroxol can promote sputum secretion *via* inhibition of MUC5AC production (Zhang et al., 2016b). It is generally accepted that MUC5AC is induced by a variety of stimuli, including interleukin (IL)-9, IL-1β, tumor necrosis factor (TNF)-α, lipopolysaccharide (LPS), neutrophil elastase, epidermal growth factor receptor (EGFR) ligands, and air pollutant (Kohri et al., 2002; Perrais et al., 2002). Therein, it has been reported that EGFR signalling cascade was documented to be a common approach by which stimuli cause MUC5AC expression (Kohri et al., 2002). EGFR is the receptor of EGF and expressed on the surface of various cells, which is a potent mitogenic factor and plays key roles in the growth, proliferation and differentiation. Hypersecretory airway diseases are associated with abnormal epithelial cell growth and proliferation, indicating that EGFR was involved in this disease. Furthermore, research has shown that EGFR is closely relevant to MUC5AC production in airway (Takeyama et al., 1999). Therefore, it may be an effective way for inhibition of EGFR/MUC5AC pathway to ameliorate the sputum obstruction.

Hepatocyte growth factor (HGF) is a highly conserved affinity ligand by specific receptor c-met, which plays a crucial role in the normal growth and development of the body. Study has suggested that HGF is manifested in the wound healing and tissue repairing in the case of embryonic development or damage to the combined organs (Zhang et al., 2013). HGF is activated and secreted from an inactive precursor by proteolytic cleavage. Once matured, it binds c-met and then exerts a multitude of bioactivities including anti-inflammatory action (Oliveira et al., 2018). Recently, it has been shown that HGF induction and reduction inflammation are involved in the tissue repair (Zhang et al., 2013). HGF inhibits interstitial inflammatory infiltration and pro-inflammatory chemokines production by disrupting nuclear factor-κB signaling in response to stress condition (Giannopoulou et al., 2008). Besides, endogenous HGF from the colon tissue can also be transferred to the body to exert some efficacy through the blood circulation (Xia et al., 2016). Although the above-mentioned evidences suggest the potential anti-inflammatory role of HGF in various conditions, whether HGF-driven airway inflammation inhibition through a gut-dependent way remains to be investigated.

Suhuang antitussive capsule (SH Capsule), one of traditional Chinese patent medicines, was approved by China Food and Drug Administration (CFDA) in 2008 and sold well with more than one billion RMB in 2018. SH Capsule has been used for the treatment of post-cold cough and cough variant asthma (CVA) in the long clinical application (Lai et al., 2018), and composed of nine traditional Chinese medicines. The fully validated botanical name is added to all botanical drug names, *i.e.*, *Ephedrae Herba* (Mahuang), *Perillae Folium* (Zisuye), *Pheretima* (Dilong), *Cicadae Periostracum* (Chantui), *Arctii Fructus* (Niubangzi), *Schisandrae Chinensis Fructus* (Wuweizi), *Peucedani Radix* (Qianhu), *Eriobotryae Folium* (Pipaye), and *Perillae Fructus* (Zisuzi) (**Table S1**). Fifty-four volatile and 50 non-volatile constituents in SH Capsule have been investigated in our laboratory by gas chromatography-mass spectrometry (GC-MS) and high performance liquid chromatography coupled with mass spectrometry (HPLC-MS) methods, respectively (Li et al., 2017; Liu et al., 2019). In addition, the fingerprints of SH Capsule have also been analyzed by HPLC (data unpublished). Furthermore, increasing evidence has shown that SH Capsule exhibits a variety of pharmacological properties, such as anti-inflammatory activity, immunomodulatory property, and attenuation airway hyperresponsiveness/remodeling (Zhang et al., 2008; Ding et al., 2016; Ou et al., 2018; Wang, 2018). Moreover, SH Capsule ameliorated CVA-associated pulmonary dysfunction *via* inhibition of endoplasmic reticulum stress and NLRP3 inflammasome activation in our previous study (Qin et al., 2019). However, there is still unknown about the definite mechanism of SH Capsule in the treatment of airway inflammation with focusing on sputum obstruction. For this, in the present study, tracheal phenol red output and mucociliary clearance tests were detected to insure the expectorant effects of SH Capsule. We investigated the inhibitory effects of SH Capsule on airway inflammation by detecting the expression of inflammatory factors and normalizing pathological changes in the airway. Furthermore, we also found that SH Capsule promoted the sputum discharge through lung inflammation-associated EGFR-ERK-MUC5AC pathway. Additionally, the present study also elucidated the mechanism through which SH Capsule increased HGF secretion in the colon, which transferred into the lung tissues through blood circulation, and thereby decreased the MUC5AC expression. As a result, SH Capsule ameliorated sputum obstruction. In conclusion, this study provided a pharmacological data for clinical use of SH Capsule, and proposed a novel mechanism by which SH Capsule was pharmacologically promising for treating sputum obstruction.

## Materials and Methods

### Materials

Fudosteine (Fud, purity ≥98%) was purchased from Feiyu Biological Technology (Nantong, Jiangsu, China). SU11274 (a selective HGF antagonist, purity ≥98%) was purchased from ApexBio (Houston, TX, USA). LPS was purchased from Sigma-Aldrich (St. Louis, MO, USA). TNF-α was purchased from Peprotech (Rocky Hill, USA). Anti-HGF (BS6234) was purchased from Bioworld (St. Paul, MN, USA). Anti-p-ERK (#4377S) and the HRP-coupled secondary antibody (#7074S or #7076S) were purchased from Cell Signal Technology (Beverly, MA, USA). Anti-MUC5AC (sc-21701) and anti-EGFR (sc-80543) were purchased from Santa Cruz Biotechnology (Santa Cruz, CA, USA). Anti-CD11b (101206) was purchased from Biolegend (San Diego, CA, USA). Anti-ERK (51068-1-AP) and anti-GAPDH (HRP-60004) were purchased from Proteintech (Chicago, IL, USA). HGF, MUC5AC, IL-6 and IL-13 Enzyme-linked Immunosorbent Assay (ELISA) kits were purchased from Elabscience Biotechnology (Wuhan, Hubei, China). Phenol red was purchased from Maikun Chemical (Shanghai, China). Rotring ink was obtained from Rotring (Hamburg, Germany).

### Sample Preparation and Analysis of SH Capsule

The drug of SH Capsule (National Drug Standard for China, YBZ00172008) was purchased and produced by Yangtze River Pharmaceutical Group Beijing Haiyan Pharmaceutical Co., Ltd (Beijing, China). The sample of SH Capsule on HPLC-MS analysis was prepared according to our previous method (Liu et al., 2019). Briefly, after removed from the shells of SH Capsule, the dried powder was ultrasonically extracted with methanol for 40 min at 60°C. The chromatogram of SH Capsule sample and mass spectra of the indicated standards were determined by HPLC-MS analysis using standardized chemicals. LC analysis was carried out by Water ACQUITY LC™ system (Waters Corp., Milford, MA, USA) coupled with a C18 column (4.6 mm × 100 mm, 3.5 µm) maintained at 28°C. The elution was performed by a mobile phase of A (water: formylic acid = 1000:1, *v/v*) and B (acetonitrile) under a gradient program of 0-60 min, gradient elution. The flow rate was 1 ml/min, and the injection volume was 2 µL. MS analysis was run with a Waters XEVO^®^ TQD system (Waters Corp.) equipped with electrospray ionization (ESI). The parameters as follows: flow rate of drying gas (N_2_), 650 L/h; desolvation temperature, 450°C; cone gas flow rate, 50 L/h; ion source temperature, 150°C; capillary voltage, 2500 V; collision gas, argon. Mass spectra were recorded across the range from 100 to 1000 m/z. Before analysis, the sample was filtered by a 0.22 µm filter membrane.

For animal experiments, the interior powder (0.45 g/capsule, equals to 4.5 g crude drug) after removed from the shells of SH Capsule was blended with appropriate saline as a working mixture for use. For *in vitro* study, the interior powder was ultrasonically extracted with 60% methanol at a 1:60 (*w/v*) ratio for 40 min at 60°C. Then the extracted solution was filtered and evaporated, and the dry extract was obtained and dissolved in dimethyl sulfoxide (DMSO) as a stock solution for use.

### Animals and Drug Administration

Male ICR mice, weighing 18-22 g, were supplied by the Laboratory Animal Center of Nanjing Qinglongshan (Nanjing, Jiangsu, China). They were housed in a specific pathogen-free animal facility on a 12 h light/dark cycle at an ambient temperature of 22 ± 1°C, and were given free access to water and food. Animal care and treatment were strictly carried out in accordance with the Provision and General Recommendation of Experimental Animals Administration Legislation of China Pharmaceutical University. All efforts were made to relieve the damage of animals and the amount of animals used. The doses chosen were based on the previous literature (Zhang et al., 2016a) and the conversion of clinical adult dosages. The clinical adult dose was three Capsules for once, three times a day according to the instructions. During service, 0.45 g/capsule of SH Capsule equals to 4.5 g crude drug, which was used to make the corresponding dosage with normal saline before administration. After acclimatization for a period of a week, they were randomized into several groups to receive saline (normal control), 3.5, 7, and 14 g/kg crude drug of SH Capsule, and the positive control 0.2 g/kg Fud (Rhee et al., 2008) *via* intragastrically (*i.g.*) or 0.18 mg/kg SU11274 *via* intraperitoneally (*i.p.*) daily for 2 weeks. The normal and control groups were given by an equal volume of normal saline in parallel. At the end of the last administration, mice were exposed to LPS (2 mg/kg) by tracheal instillation. After treatment for 24 h, mice were anesthetized and subjected to alveolar lavage, and the trachea and lung tissues of the mice were collected for other measurement.

### Tracheal Phenol Red Output Test

The procedure of tracheal phenol red output was performed by the study as previously described (Engler and Szelenyi, 1984; Zhang et al., 2016b). Briefly, mice were randomized into 5 groups according to weight difference, which received saline (normal control), 3.5, 7 and 14 g/kg SH Capsule, and the positive control 0.2 g/kg Fud for only once, respectively. Then, assessment of tracheal phenol red output was carried out. After 30 min of administration with a single dose of the test drugs for each mouse, they were *i.p.* received phenol red solution (5% in saline solution, 0.2 ml/20 g body weight). About 30 min later, mice were anaesthetized as killed, then dissected away other adjacent organs and put the trachea (stem bronchi) into 2 ml of normal saline at 4°C overnight. The supernatant was determined by using a microplate reader (Biotek Epoch, Winooski, VT, USA) at 558 nm. The concentration of phenol red was calculated by the standard curve according to the previous reported (Engler and Szelenyi, 1984).

### Mucociliary Clearance

The ink solution was diluted to 5% (rotring ink solution was 100%) with saline and centrifuged at 1000 g for 10 min. The supernatant was harvested for this study. After acclimatization for a period of a week, mice were randomly divided into 5 groups. They were received saline (normal control), 3.5, 7 and 14 g/kg SH Capsule, and the positive control 0.2 g/kg Fud once daily for 2 weeks. After 30 min of the last administration, mice were subjected to the supernatant of ink solution (30 µL/20 g body weight) by tracheal instillation. After another 30 min, the broncho alveolar lavage fluid (BALF) was collected (Deng et al., 2006). BALF was centrifuged for 10 min at 1000 g, and assayed by a microplate reader (Biotek Epoch, Winooski, VT, USA) at 500 nm.

### Histological Analysis and Immunohistochemistry

Mice were euthanized and killed by cervical dislocation. The tracheas were dissected from mice and fixed with 4% (*w/v*) paraformaldehyde, embedded in paraffin. After embedding, they were sectioned at 5 µm thickness and stained with hematoxylin and eosin (H&E) for general histological examination, and the lung was incubated with 0.5% periodic acid solution (PAS) and stained with Schiff’s reagent for general histological examination. For immunohistochemistry, the lung and trachea tissues were obtained from mice, and immersed in fixative 10% formalin. They were processed for staining by indicated primary antibody following the manufacturer’s instructions using the standard protocols. Images were captured by an optical microscope (Leica DMi8, Wetzlar, Heessen, Germany).

### Cell Culture

Human colon carcinoma cells (HT-29 cells) were purchased from the cell bank of the Chinese Academy of Sciences (Shanghai, China), which were respectively maintained in Dulbecco’s Modified Eagle Medium/F12 medium (DMEM/F12) supplemented with 10% (*v/v*) fetal bovine serum (FBS, Biological Industries, Kibbutz Beit-Haemek, Israel) under a humidified atmosphere of 5% CO_2_ humidified air at 37°C.

### Cell Viability Assay

The effects of SH Capsule on HT-29 cell viability were measured by the 3-(4,5-dimethylthiazolyl-2)-2,5-diphenyltetrazolium bromide (MTT) assay. HT-29 cells were seeded in 96-well plates at a density of 2 × 10^4^ cells/ml, and treated with SH Capsule in the indicated concentration for 24 h. Then, the cells were incubated with MTT solution (0.5 mg/ml) for 4 h at 37°C, and the formazan crystals were dissolved in DMSO (150 µL/well). The absorbance was measured with a microplate reader (Biotek Epoch, Winooski, VT, USA) at 490 nm.

### Flow Cytometry Analysis

The protocol of flow cytometric analysis was carried out by standard procedures. Briefly, BALF was harvested and centrifuged at 1000 g for 10 min. Then they were stained with the specific antibodies CD11b (Biolegend, San Diego, CA, USA) for 30 min at 37°C in dark and assessed by flow cytometry (Attune^®^ NxT, Thermo, Waltham, MA, USA).

### Quantitative-Polymerase Chain Reaction Assay

Total RNA was isolated from the tissues homogenates and HT-29 cells by using Trizol reagent (Takara, Otsu, Japan). According to the manufacturer’s protocol, cDNA synthesis was carried out by commercial cDNA synthesis kits (Vazyme Biotech, R223-01), and subjected to quantitative PCR using SYBR Green Premix (Vazyme Biotech, Q141-02/03) with a StepOne Real-Time PCR System (Applied Biosystems). Sequences of the primers are listed in **Table S2**.

### Enzyme-Linked Immunosorbent Assay

The levels of IL-6, IL-13, MUC5AC, and HGF in the supernatant were quantified and determined by using ELISA kits according to the manufacturer’s instructions. The absorbance was read by a microplate reader (Biotek Epoch, Winooski, VT, USA) at 450 nm.

### Western Blotting Analysis

Total proteins from cells or tissue samples were lysed in lysis buffer, which were determined using a BCA assay (Beyotime, Shanghai, China). Then, cell lysate was loaded each well into 10% SDS-PAGE gels and transferred to a PVDF membrane (IPVH00010, Millipore, Bedford, MA, USA). The membranes were blocked in 5% non-fat milk in TBST for 2 h at room temperature and incubated with the indicated primary antibodies at 4°C overnight. After incubation with the secondary antibody at room temperature for 2 h, signals were performed by using a Tanon 5200 Multi chemiluminescent substrate system (Tanon, Shanghai, China). The values of band intensities were quantized by Image-ProPlus 6.0 software.

### Immunofluorescence

HT-29 cells (8 × 10^5^ cells/ml) were seeded onto the cover slips, and treated with SH Capsule (100 µg/ml) in the presence or absence of TNF-α (50 ng/ml) for 24 h. After treatment, they were fixed by 4% paraformaldehyde for 20 min, permeabilized with Triton X-100 (0.5%) for 20 min and blocked with 3% BSA for 1.5 h. After washing with PBS twice, they were stained with anti-HGF antibody overnight at 4°C, and then with specific goat-anti rabbit IgG-Alexa 488 secondary antibody for 1.5 h. Subsequently, they were counterstained with DAPI (Beyotime) and imaged with a fluorescence microscope (Leica DMi8).

### Statistical Analysis

Data are identified as the mean ± standard error of the mean (SEM). Statistics were analyzed by using the two-tailed Student’s *t* tests or one-way ANOVA with Dunnett’s multiple comparisons test. *p*-values <0.05 was considered statistically significant.

## Results

### SH Capsule Promotes the Tracheal Phenol Red Output and Mucociliary Clearance in Mice

To ensure the quality of SH Capsule in the present study, we have determined and identified some major chemical and effective components in SH Capsule by using LC-MS analysis based on the standard reference compounds. The chemical components of SH Capsule were shown in **Figure S1**, *i.e.*, ephedrine, arctiin, arctigenin, schisandrin, schisandrol B, and schisandrin B, which have been described in our previous works (Li et al., 2017; Liu et al., 2019; Qin et al., 2019). This gives a scientific data for SH Capsule to serve as a stable subject of pharmacological experiment in our study.

**Figure 1:**
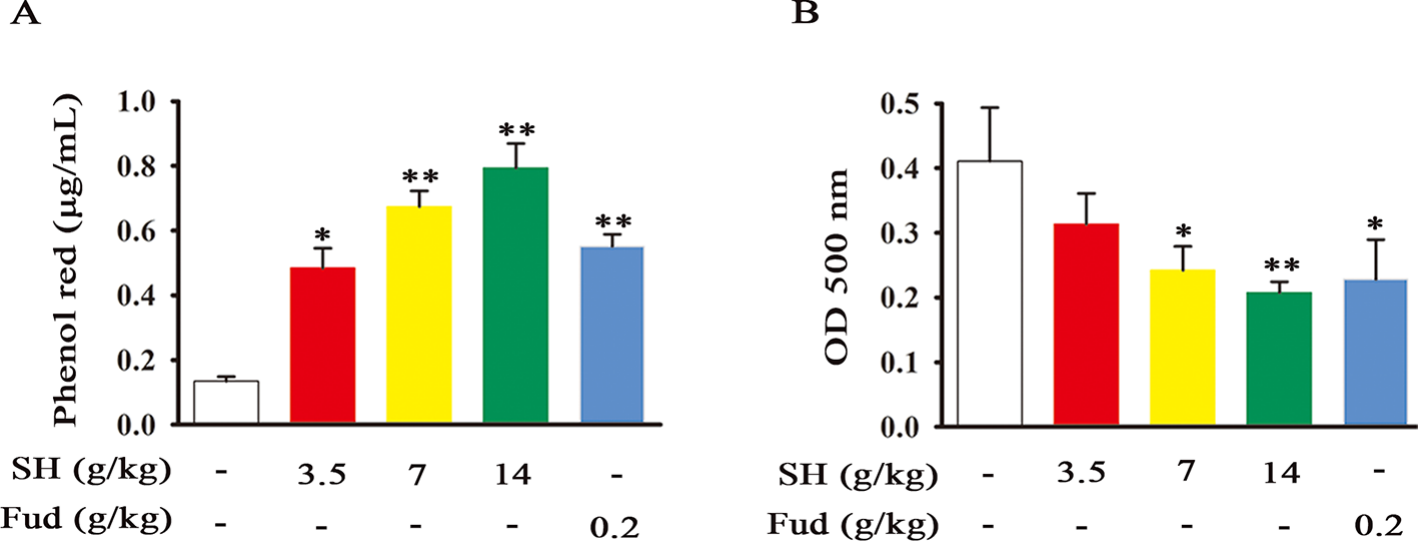
SH Capsule Promotes the Tracheal Phenol Red Output and Mucociliary Clearance in Mice. ICR mice were dosed with SH Capsule (3.5, 7, 14 g/kg) and Fud (0.2 g/kg) for once. **(A)** The phenol red output and **(B)** mucociliary clearance were detected. Data were presented as the mean ± SEM (n = 6). *p < 0.05, **p < 0.01 *vs.* the untreated group.

The tracheal phenol red excretion test is a simple and effective method to investigate the effects of agents on tracheobronchial secretion (Engler and Szelenyi, 1984). To assess the potential action of SH Capsule on promoting tracheal secretion, phenol red output was examined after intraperitoneal injection of phenol red solution. As shown in **Figure 1A**, SH Capsule dose-dependently increased the solution color and phenol red concentration in normal mice. Fud also showed a similar effect as SH Capsule. Besides, mucociliary clearance is impaired in respiratory disease patients due to a reduction in ciliary epithelial cells or a change in mucus production, or both (Wanner, 1977). Accordingly, the effects of SH Capsule on mucociliary clearance were determined in BALF. Because the color of BALF was black in the untreated group, the OD value was high. Whereas, the OD values of SH Capsule and Fud treatment groups were reduced (**Figure 1B**), indicating that SH Capsule enhanced the mucociliary clearance to exhaust sputum. Besides, during the whole experiments, we inspected the body weights of mice and the weight of standard chow as well as mental state of mice daily for two weeks. In contrast with normal group, there were no differences between them. These data showed that SH Capsule possessed the function of strengthening the tracheal phenol red output and ciliary movement.

### SH Capsule Reduces the MUC5AC Expression in BALF and Lung Tissues of LPS-Exposed Mice

**Figure 2:**
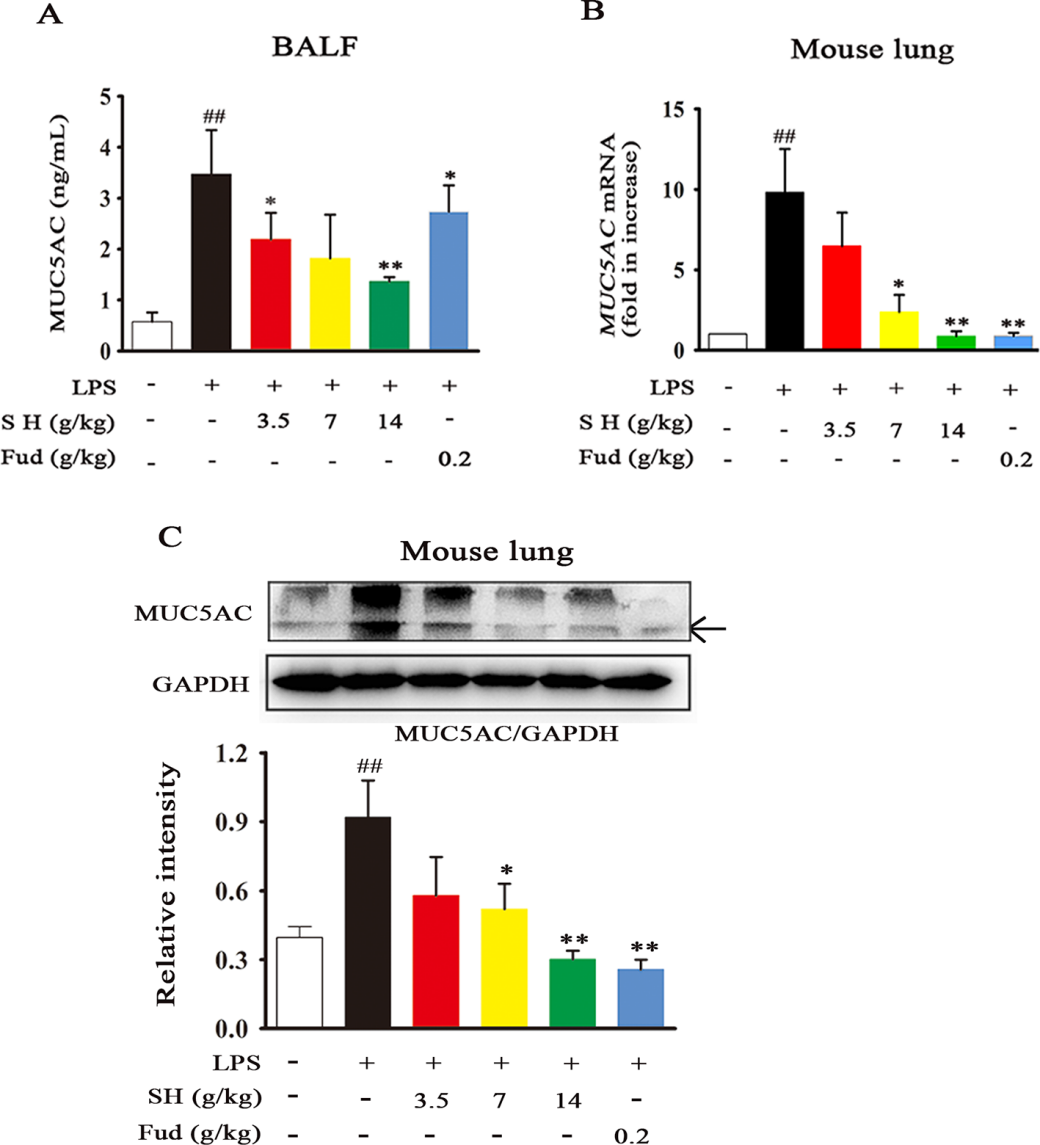
SH Capsule Reduces the MUC5AC Level in BALF and Lung Tissues of LPS-Exposed Mice. ICR mice were dosed with SH Capsule (3.5, 7, 14 g/kg) and Fud (0.2 g/kg) for two weeks. The lung tissues and BALF from LPS-exposed mice were collected after LPS exposure for 24 h. **(A)** The level of MUC5AC in BALF was measured by ELISA. **(B)** The mRNA expression of *MUC5AC* was determined by Q-PCR. **(C)** MUC5AC expression in the lung tissues was detected by western blotting. Data were shown as the mean ± SEM (n = 6). ^##^ *p* < 0.01 *vs.* the untreated group. *p < 0.05, **p < 0.01 *vs.* LPS only treatment group.

As a key regulation of sputum secretion processing, the airway MUC5AC level was analyzed. As shown in **Figure 2A**, LPS treatment caused a dramatic increase in the MUC5AC level of BALF, whereas this alternation was restored by administration of SH Capsule. Subsequently, we further observed the effects of SH Capsule on the mRNA and protein levels of MUC5AC in lung tissues. As presented in **Figures 2B, C**, LPS challenges induced MUC5AC increase in both mRNA and protein levels, whereas SH Capsule dose-dependently attenuated the increased mRNA and protein levels of MUC5AC in the lung tissues of LPS-exposed mice. Consistently, the positive control drug, Fud, also reduced the MUC5AC mRNA and protein level (**Figures 2A–C**). These results indicated that SH Capsule may promote the sputum discharge by inhibiting MUC5AC expression.

### SH Capsule Alleviates LPS-Induced Pulmonary Inflammation in Mice

**Figure 3:**
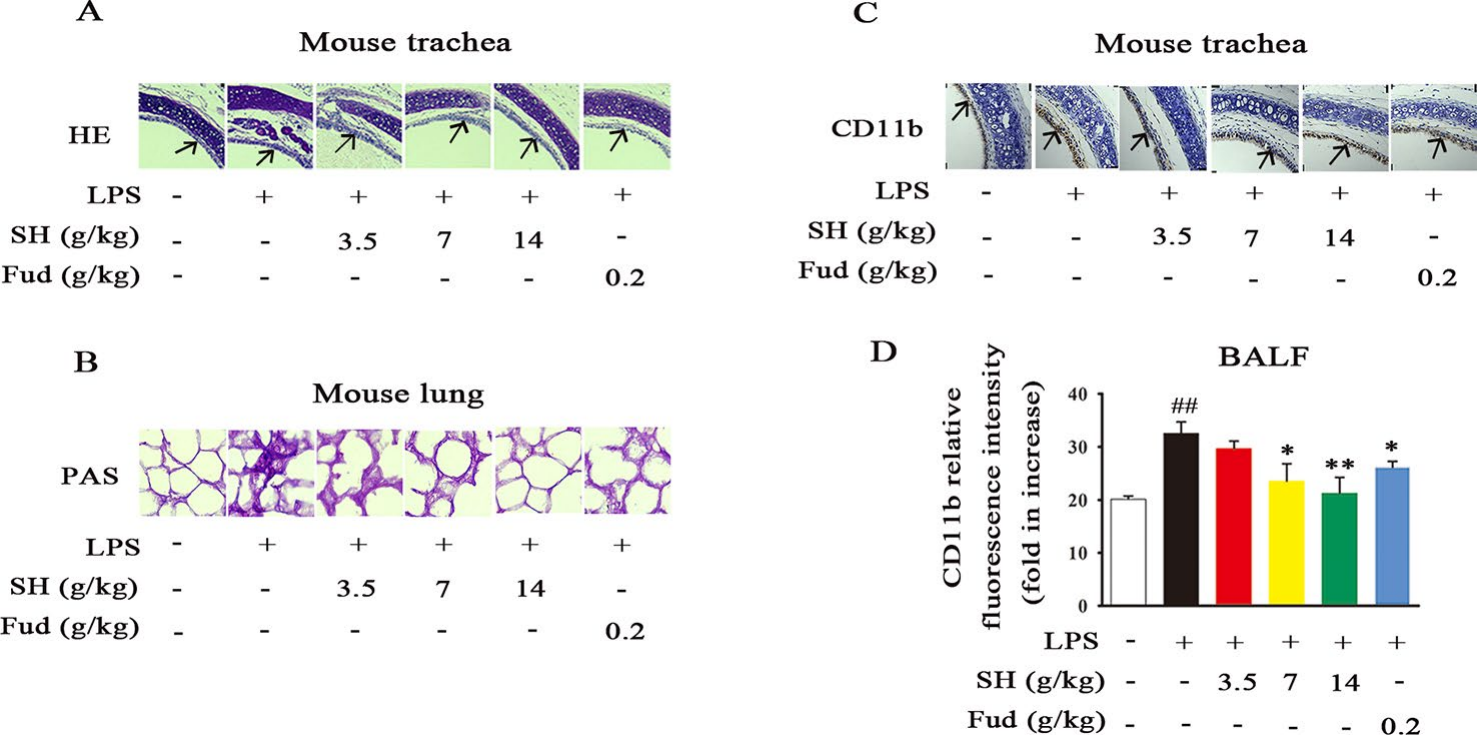
SH Capsule Alleviates LPS-Induced Pulmonary Inflammation in Mice. The BALF, trachea and lung tissues were collected after LPS exposure for 24 h. **(A)** Pathological changes in trachea tissues were determined by HE staining. **(B)** The level of glycoprotein in the lung tissues was measured by PAS staining. **(C)** CD11b in trachea was detected by immunohistochemistry. **(D)** CD11b level in BALF was measured by flow cytometry. Data were shown as the mean ± SEM (n = 6). ^##^ *p* < 0.01 *vs.* the untreated group. *p < 0.05, **p < 0.01 *vs.* LPS only treatment group.

Increasing evidence has shown that inflammation may contribute to disordering the sputum discharge (Imamura et al., 2004). When inflammation occurs in the lung, it will cause airway wall thickening/narrowing, mucosal movement weaken and adhesion protein secretion increase. Due to the paramount roles of inflammation in sputum obstruction, we then evaluated the effects of SH Capsule on airway and lung inflammation by the HE and PAS staining. As demonstrated in **Figure 3A**, LPS treatment induced the germination of inflammation, indicated by the increase in the parenchymal distortion and inflammatory cell infiltration in the trachea. Whereas, SH Capsule effectively decreased these changes. Similar to the regulative roles of SH Capsule in inflammation, SH Capsule showed an attenuated mucus secretion in the lung tissues of mice by PAS staining (**Figure 3B**). Meantime, we further examined an inflammatory macrophage marker CD11b in the trachea by immunohistochemistry staining. As shown in **Figure 3C**, SH Capsule remarkably inhibited macrophage CD11b overexpression in the trachea of LPS-exposed mice. Coincidentally, flow cytometry analysis showed that SH Capsule normalized macrophage CD11b level in BALF (**Figure 3D**), indicative of the necessary of SH Capsule action in the initiation of inflammatory response. Fud also showed a similar effect as SH Capsule. These results demonstrated that SH Capsule mitigated inflammatory responses in LPS-exposed mice.

### SH Capsule Inhibits the Elevation of Inflammatory Mediators in Both Lung Tissues and BALF

**Figure 4:**
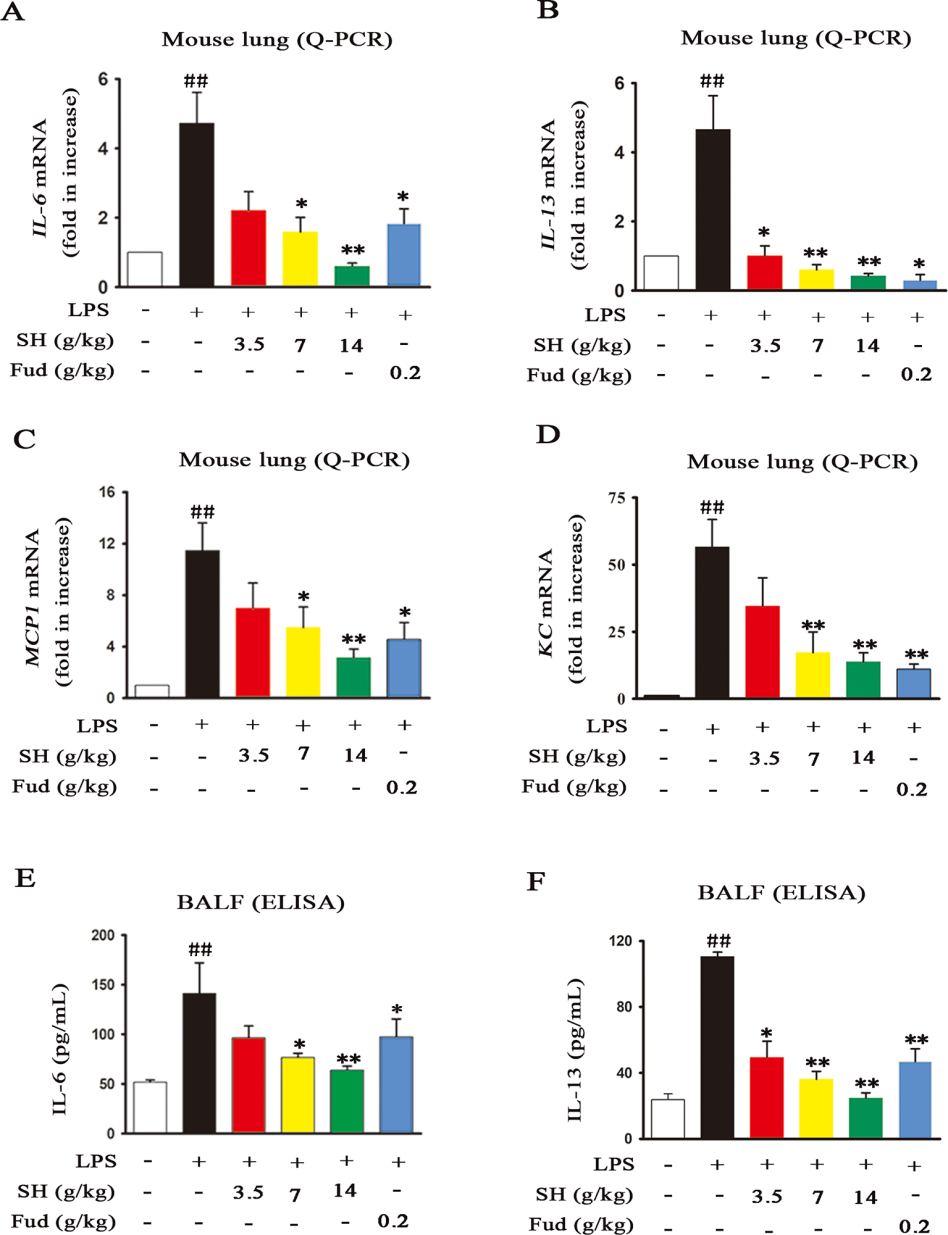
SH Capsule Inhibits the Elevation of Inflammatory Mediators in both Lung Tissues and BALF. The lung tissues and BALF were collected after LPS exposure for 24h. **(A**–**D)** The mRNA levels of *IL-6*, *IL-13*, *MCP-1* and *KC* in the lung tissues were measured by Q-PCR analysis. **(E**–**F)** The protein levels of IL-6 and IL-13 in BALF were detected by ELISA. Data were presented as the mean ± SEM (n = 6). ^##^ *p* < 0.01 *vs.* the untreated group. *p < 0.05, **p < 0.01 *vs.* LPS only treatment group.

Accompanied with pulmonary inflammation, inflammatory mediators will enormously congregate in the lung. In order to evaluate the effects of SH Capsule on inflammatory mediators, mRNA levels of *IL-6*, *IL-13*, *MCP-1* and *KC* in the lung tissues were measured by using Quantitative-Polymerase Chain Reaction (Q-PCR) analysis. As shown in **Figures 4A–D**, the mRNA expressions of *IL-6*, *IL-13*, *MCP-1* and *KC* were statistically increased after LPS challenge. However, administration of SH Capsule dose-dependently suppressed the expression of these genes in LPS-challenged mice. Because mRNA expression is a precursor event for protein level, we next estimated the effects of SH Capsule on LPS-induced inflammatory cytokines secretion. As expected, SH Capsule observably decreased the expression levels of inflammatory cytokines IL-6 and IL-13 in response to LPS challenge (**Figures 4E, F**). Similarly, Fud also inhibited the mRNA and protein expression of inflammatory mediators. Collectively, SH Capsule alleviated lung inflammation of LPS-exposed mice *via* reduction of inflammatory mediators expression.

### Effects of SH Capsule on the EGFR Expression and ERK Phosphorylation in the Lung Tissues of LPS-Exposed Mice

**Figure 5:**
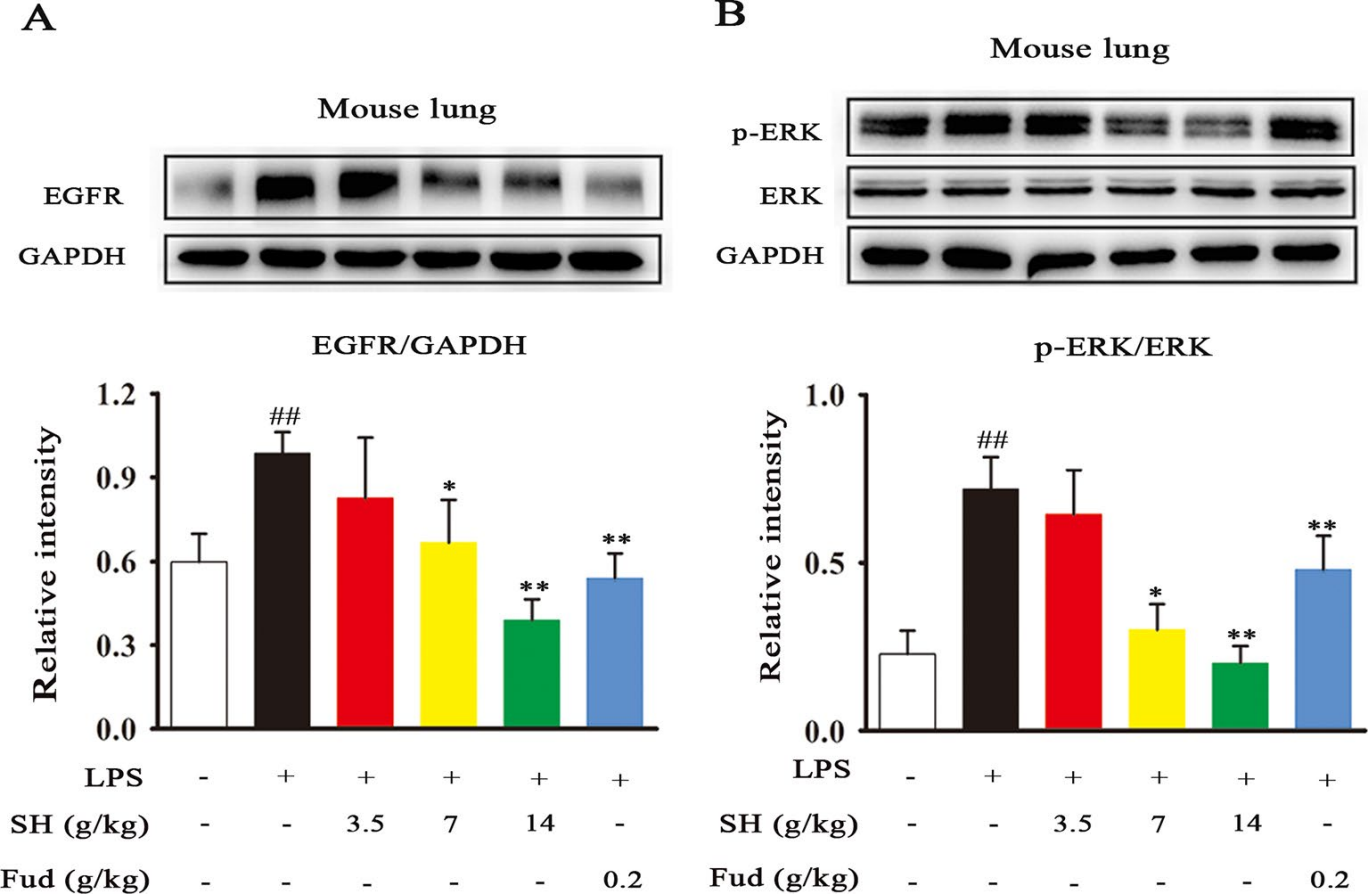
SH Capsule Suppresses the EGFR Expression and ERK Phosphorylation in the Lung Tissues of LPS-Exposed Mice. **(A)** The expression of EGFR in the lung tissues was determined by western blotting. **(B)** ERK phosphorylation was examined in the lung tissues by western blotting. Data were presented as the mean ± SEM (n = 6). ^##^ *p* < 0.01 *vs.* the untreated group. *p < 0.05, **p < 0.01 *vs.* LPS only treatment group.

To know the special potential pathway of SH Capsule in the involvement of inflammation-mediated sputum obstruction, we subsequently investigated the effects of SH Capsule on regulating the expression of EGFR and ERK, which play key roles in the regulation of inflammation and MUC5AC (Kohri et al., 2002). As shown in **Figures 5A, B**, LPS challenge markedly up-regulated the EGFR production and increased ERK phosphorylation. Impressively, EGFR level and ERK phosphorylation were effectively reduced after SH Capsule treatment (**Figures 5A, B**), suggesting that EGFR/ERK signal was required for the roles of SH Capsule in anti-inflammation. As expected, Fud also decreased EGFR expression and ERK phosphorylation in response to LPS challenge. These results indicated that SH Capsule inhibited LPS-induced EGFR expression and ERK activation, demonstrating its anti-inflammatory action through EGFR/ERK pathway.

### SH Capsule Promotes HGF Generation in HT-29 Cells

**Figure 6:**
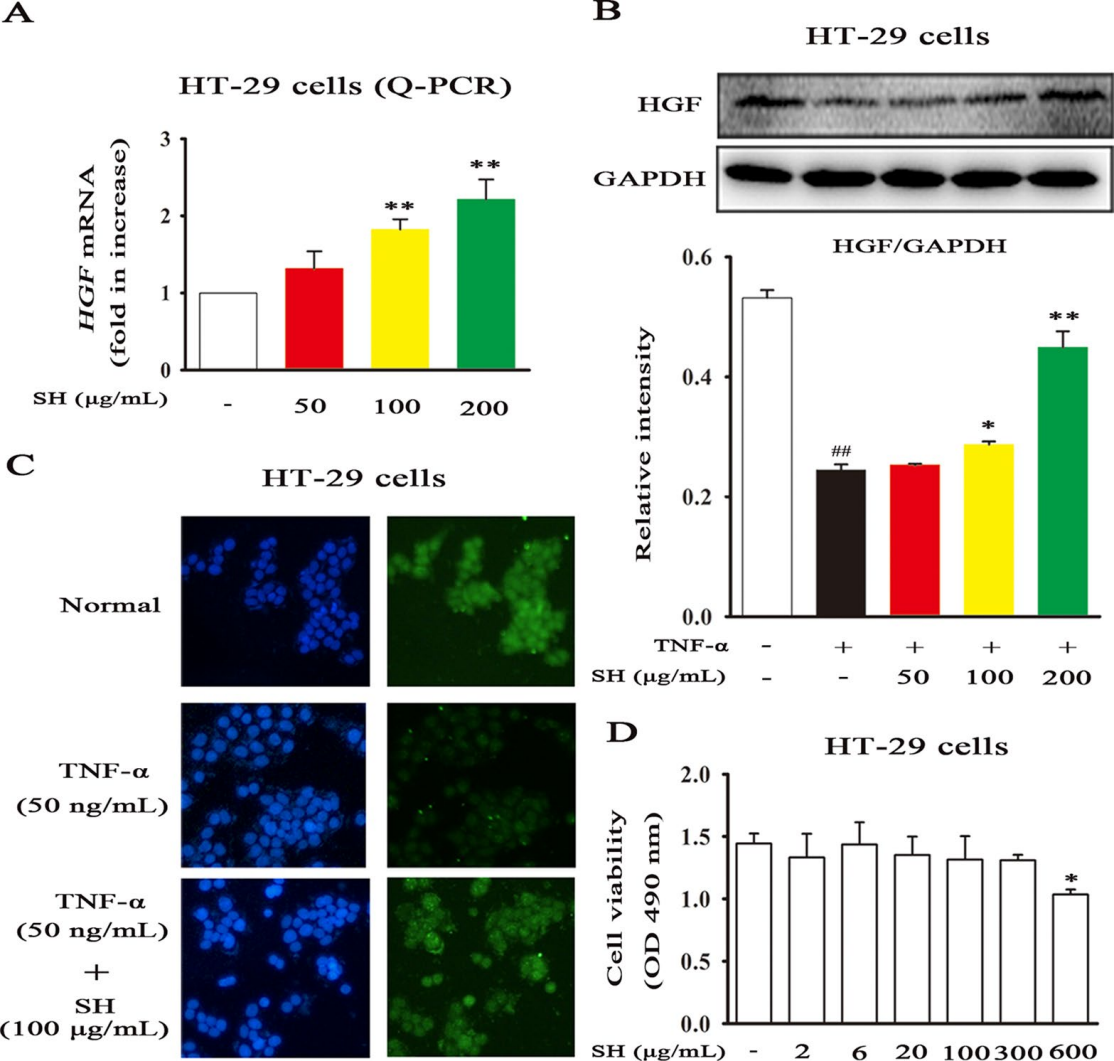
SH Capsule Promotes HGF Generation in HT-29 Cells. HT-29 cells were treated with SH Capsule (50, 100, 200 μg/mL) in the presence or absence of TNF-α (50 ng/ml). **(A)** The mRNA expression of *HGF* was determined by Q-PCR. **(B)** The protein expression of HGF was detected by western blotting. **(C)** HGF level was detected by immunofluorescence staining. **(D)** HT-29 cell viability was determined by MTT assay. Data were presented as the mean ± SEM of three independent experiments. ^##^ *p* < 0.01 *vs.* the untreated group. *p < 0.05, **p < 0.01 *vs.* untreated group or TNF-α only treatment group.

Recent study has shown that HGF plays a vital role in respiratory diseases, such as, lung cancer, inflammation and fibrosis (Xia et al., 2016). Then, to examine the stimulative effects of SH Capsule on HGF production in HT-29 cells, the mRNA and protein levels of HGF were assessed. SH Capsule concentration-dependently increased the mRNA expression of *HGF* in HT-29 cells (**Figure 6A**). Coincidentally, immunoblot showed that SH Capsule significantly promoted HGF secretion in TNF-induced HT-29 cells (**Figure 6B**). Conformably, immunofluorescence of HGF also ensured this phenomenon (**Figure 6C**). These results also further demonstrated SH Capsule promoted gut-derived HGF production. To observe whether SH Capsule has an effect on cytotoxicity-mediated HT-29 cells on the results described above, the cell viability was measured. As shown in **Figure 6D**, SH Capsule did not affect the viability of HT-29 cells at concentration from 2 to 300 µg/ml. These finding showed that SH Capsule promoted HGF secretion without cytotoxicity in HT-29 cells.

### The HGF Inhibitor SU11274 Abrogates the Effects of SH Capsule on MUC5AC Levels in LPS-Induced Lung Tissues

**Figure 7:**
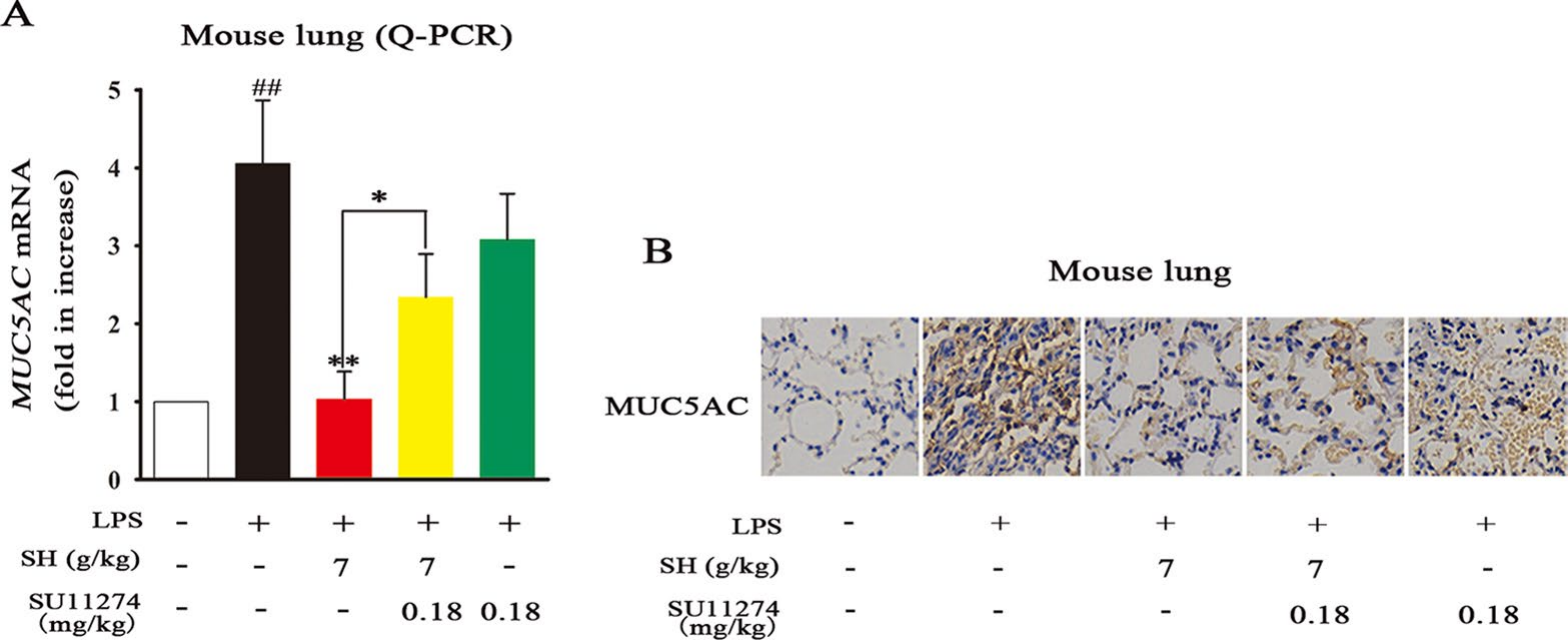
The HGF Inhibitor SU11274 Abrogates the Effects of SH Capsule on MUC5AC Levels in LPS-Induced Lung Tissues. The lung tissues were collected after LPS exposure for 24 h. **(A)** The mRNA expression of *MUC5AC* in the lung tissues was detected by Q-PCR. **(B)** The expression of MUC5AC was examined by immunohistochemistry staining. Data were presented as the mean ± SEM (n = 6). ^##^ *p* < 0.01 *vs.* the untreated group. *p < 0.05, **p < 0.01 *vs.* LPS only treatment group or the indicated group.

To verify whether or not HGF production plays a strategic role in the ameliorative effects of SH Capsule on MUC5AC levels, a specific HGF receptor inhibitor SU11274 was taken in this study. As shown in **Figures 7A, B**, LPS treatment effectively induced airway MUC5AC increase, in contrast, SH Capsule was indeed able to prevent LPS-induced mRNA and protein levels of MUC5AC in mice. SU11274 had no effect on MUC5AC expression, but it almost completely reversed the ameliorative effects of SH Capsule on MUC5AC expression, suggesting that HGF production is essential for the inhibitory roles of SH Capsule in MUC5AC expression. This finding demonstrated that HGF was involved in the effects of SH Capsule on promoting sputum discharge.

### SH Capsule Selectively Promotes the HGF Secretion in the Colon of Mice

**Figure 8:**
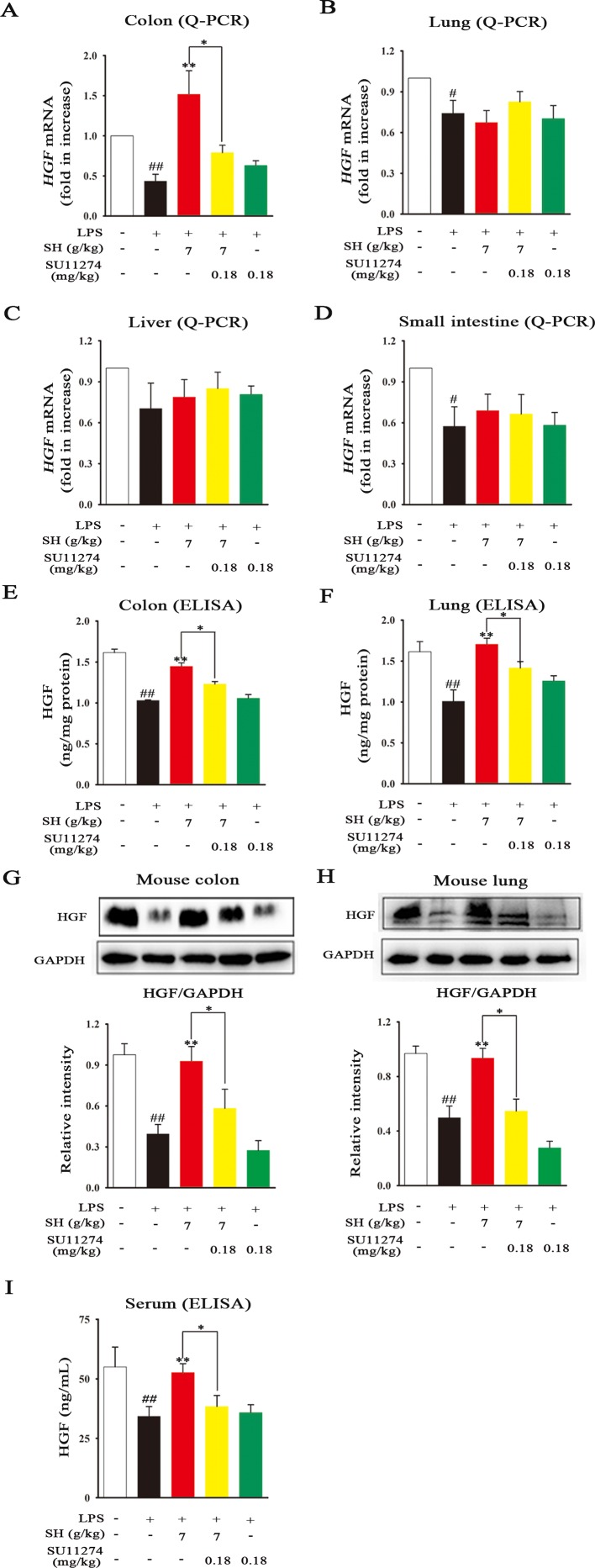
SH Capsule Selectively Promotes the HGF Secretion in the Colon of Mice. The colon, lung, liver and small intestine tissues were collected after LPS exposure for 24 h. **(A**–**D)** The mRNA expression of *HGF* in the colon, lung, liver and small intestine tissues was detected by Q-PCR. **(E**–**H)** The protein levels of HGF in the colon and lung tissues were measured by ELISA and western blotting. **(I)** The level of HGF in serum was determined by ELISA. Data were presented as the mean ± SEM (n = 6). ^#^ *p* < 0.05, ^##^ *p* < 0.01 *vs.* the untreated group. *p < 0.05, **p < 0.01 *vs.* LPS only treatment group or the indicated group.

Emerging evidence demonstrates that endogenous factor HGF mainly originates from gut, which can contribute to repair the lung-associated diseases (Guan et al., 2018). To clarify which sites that SH Capsule promoted HGF secretion, the mRNA expression of *HGF* in the colon, lung, liver and small intestine was detected by Q-PCR. As shown in **Figures 8A–D**, LPS challenge led to a significant reduction in HGF mRNA levels. By contrast, oral administration of SH Capsule markedly increased the mRNA levels of *HGF* in the colon but not in the lung, liver and small intestine of LPS-exposed mice. However, SU11274 almost abrogated the effects of SH Capsule on *HGF* mRNA level. Astonishingly, the results from both ELISA and immunoblot analysis showed that the protein levels of HGF in the colon and lung were all dramatically increased by SH Capsule treatment (**Figures 8E–H**). SU11274 also deleted the effects of SH Capsule on HGF secretion. We wondered whether HGF secretion in the colon could be transported to the lung through blood circulation. As shown in **Figure 8I**, SH Capsule effectively increased HGF level in serum, which might be released in the colon. Similarly, SU11274 deteriorated the effects of SH Capsule on HGF production, clearly indicative of the important roles of SH Capsule in HGF secretion from the gut. These findings supported our hypothesis that colon-derived HGF secretion through the blood transport was essential for the effects of SH Capsule on promoting sputum discharge.

## Discussion

**Figure 9:**
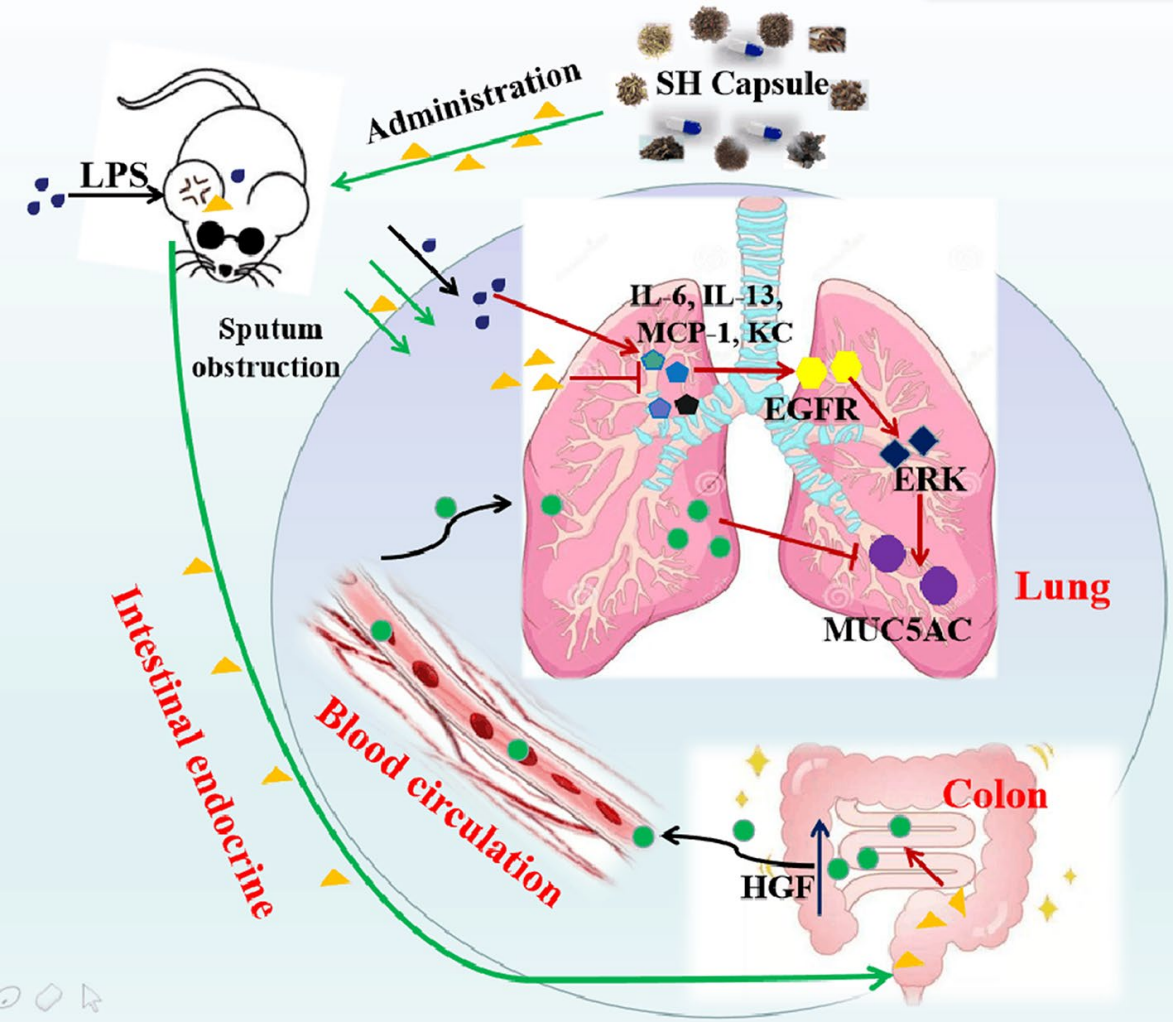
The Proposed Pathway for SH Capsule Action in the Inhibition of Sputum Obstruction. SH Capsule promotes HGF secretion in intestinal endocrine, transporting to the lung through blood circulation, and ameliorates airway inflammation-associated sputum obstruction by reduction of MUC5AC expression through EGFR/ERK pathway.

The contribution of airway inflammation to mucin hypersecretion will facilitate the sputum accumulation, which greatly affects the breathing, dieting, sleeping, and increases the burden on individuals/society. Hence, discovering an agent for the prevention and treatment of inflammation-associated sputum obstruction is urgently needed. In the present study, we demonstrated that SH Capsule, one of traditional Chinese patent medicines, ameliorated the sputum obstruction in LPS-challenged mice through promoting HGF secretion in an intestinal endocrine-dependent manner. This study enables us to comprehend a novel mechanism route and pharmacological role of SH Capsule action in the pathogenesis of airway inflammation-mediated mucous hypersecretion. The proposed schema of the pharmacological underlying mechanism of SH Capsule is shown in **Figure 9**.

As well known, many plants and plant-derived formulations have been used as functional foods and traditional Chinese medicines characterized by multi-components and multi-targets (Qiu, 2015). SH Capsule is used for the treatment of post-cold cough and cough variant asthma in clinical, which is originated from a classic traditional Chinese medicine prescription under the theoretical system of Chinese medicine. In this study, we found that SH Capsule increased tracheal phenol red excretion and mucociliary clearance in normal mice (**Figure 1**), well clarifying that SH Capsule effectively promoted the discharge of sputum. Consistently, histological examination of the trachea tissues by PAS staining also demonstrated this phenomenon (**Figure 3**). Additionally, the overproduction of mucin protein is one of the main reasons for the increase in sputum viscosity. Among the major gel-forming mucins in the airway, MUC5AC has been extensively discussed one due to its role in mucus hypersecretion (Williams et al., 2006). Our study found that SH Capsule dose-dependently reduced the mRNA and protein level of MUC5AC in the lung tissues of LPS-induced mice (**Figure 2**), which proved that SH Capsule could decrease the viscosity of sputum. These results also directly and indirectly explained why SH Capsule increased tracheal phenol red excretion and mucociliary clearance in mice. Apart from airway narrowing and reduction of ciliary movement, LPS-induced airway inflammation also plays a crucial role in mucus hypersecretion-mediated sputum obstruction (Yang et al., 2017). A causal link has been established between the uncontrolled inflammation and mucin hypersecretion during respiratory diseases (Chen et al., 2014). Besides, chronic persistent inflammation also contributes to the occurrence of pulmonary fibrosis and lung cancer. It is widely acknowledged that LPS binds to the TLR4 receptor, which is essential for inducing inflammation signaling pathway (Okuyama et al., 2017). Once activated, it turned out that a multitude of pro-inflammatory cytokines will be released in the surrounding tissues environment. In the present study, we provided the evidences for the relationship between airway inflammation occurrence and mucin secretion through which SH Capsule inhibited LPS-induced MUC5AC overexpression. Based on LPS-induced airway inflammation events, the results showed that SH Capsule effectively prohibited the expression of pro-inflammatory mediators, including *IL-6, IL-13, MCP-1* and *KC* in the lung tissues of LPS-induced mice (Zhang et al., 2016b) (**Figure 4**). Increasing evidence demonstrates that airway inflammation is tightly associated with ERK1/2 activation, which may increase sputum viscosity (Hou et al., 2015; Shin et al., 2016). We further found that SH Capsule inhibited the EGFR expression and ERK phosphorylation in the lung tissues of LPS-induced mice (**Figure 5**). This result indicated that SH Capsule attenuated airway inflammation and mucin hypersecretion through EGFR/ERK/MUC5AC signaling pathway, which may be one of its molecular mechanisms.

The intestinal tract, one of the largest endocrine organs, is a major local site which can secrete many growth factors and pleiotropic cytokines (Porowski et al., 2009). Among them, HGF is one of the antifibrotic factor agents, involving in the process of tissue repair and anti-inflammatory activity (Zhang et al., 2013). Besides, recent study has shown that exogenous HGF contributes to lung repair in the model of lung injury (Xia et al., 2016; Guan et al., 2018). In this study, we found that SH Capsule concentration-dependently increased HGF secretion in HT-29 cells (**Figure 6**). This finding prompted us to postulate that SH Capsule promoted the endogenous HGF from the intestinal tract. Then, HGF came into the blood circulation and transferred into the lung tissues, displaying anti-inflammatory activity. In order to verify this hypothesis, the effects of SH Capsule on HGF secretion in the lung, liver, small intestine and colon of LPS-induced mice were investigated. The result showed that SH Capsule did not affect the mRNA expressions of *HGF* in the lung, liver, small intestine, but significantly increased the mRNA expression of *HGF* in the colon tissues of LPS-induced mice (**Figure 8**). As expected, SH Capsule observably increased the protein levels of HGF in the colon, serum and lung tissues of LPS-induced mice. This seemed a incompatible alteration about SH Capsule-treated *HGF* mRNA and protein levels in the serum and lung tissue of mice. In combination with the characteristics of traditional Chinese medicine and correlative pharmacokinetic knowledge, we speculated that SH Capsule increased HGF secretion in blood circulation and lung tissues, which was originated from the colon tissue of mice.

In accordance with above-mentioned findings, the present study indicated that the specific HGF receptor inhibitor SU11274 markedly reversed the effects of SH Capsule on HGF secretion and MUC5AC level (**Figure 7**). This result demonstrated that decreasing MUC5AC level in the lung tissues of LPS-exposed mice treated by SH Capsule was secondary to promote HGF secretion. SH Capsule may inhibit inflammation and promote lung tissue repair *via* increasing endogenous HGF production, especially in the colon tissues. Although HGF is firstly noted in a mitogenic protein in the hepatocyte of rats, it’s subsequently used in the mucosal repair during inflammatory bowel disease (Ortega-Cava et al., 2002). In this study, HGF possesses anti-inflammatory effects by reducing the expression of inflammatory mediators. Hence, when HGF arrived into the lung, it can suppress the lung inflammation and promote lung repair (Panganiban and Day, 2011). These findings showed that HGF is indeed involved in the pathogenesis of airway inflammation and lung injury.

To ensure the quality of SH Capsule in the present study, we have analyzed some key effective chemical components using LC-MS (**Figure S1**), and we have also identified over one hundred constituents in SH Capsule in our previously works (Li et al., 2017; Liu et al., 2019), which suggests that SH Capsule was completely in conformity with our pharmacological study. Further study is needed to figure out which chemical components are responsible for improving sputum obstruction.

In conclusion, this study demonstrates that SH Capsule ameliorates airway inflammation-associated sputum obstruction induced by LPS in mice through promoting HGF secretion *via* an intestinal endocrine-dependent pathway. It is the first time to reveal that the mechanism of SH Capsule on promoting the expectoration. Our work provides new pharmacological data for clinical use of SH Capsule, and proposed a novel mechanism by which SH Capsule was pharmacologically promising for treating sputum obstruction.

## Data Availability Statement

All datasets generated for this study are included in the article/**Supplementary Material**.

## Ethics Statement

The animal study was reviewed and approved by The Provision and General Recommendation of Experimental Animals Administration Legislation of China Pharmaceutical University.

## Author Contributions

XT and NT performed the research, analyzed the data, and wrote the manuscript. RL, WQ and CG contributed to animal experiments and cell culture. YJ and XW contributed to the preparation of SH Capsule. ZW, DC and NT designed and conceptualized the research, and revised the manuscript. All authors read and approved the final manuscript.

## Funding

This work was supported by the Fund of State Key Laboratory of Natural Medicines of China Pharmaceutical University (3144060028); the Program for Innovative Research Team of Jiangsu Province; the National New Drug Innovation Major Project of the Ministry of Science and Technology of China (2017ZX09309027); the Graduate Student Innovative Foundation of Zhejiang Huahai Pharmaceutical Co., Ltd. (CX17S-010HH); the Postgraduate Research and Practice Innovation Program of Jiangsu Province (KYCX19_0693); and the “Double First-Class” University Project from China Pharmaceutical University (CPU2018GF05).

## Conflict of Interest

YJ and DC were employed by Yangtze River Pharmaceutical Group Beijing Haiyan Pharmaceutical Co., Ltd.

The remaining authors declare that the research was conducted in the absence of any commercial or financial relationships that could be construed as a potential conflict of interest.

## Abbreviations

DMSO, dimethyl sulfoxide; EGFR, epidermal growth factor receptor; ERK, extracelluar signal-regulated kinase; LPS, lipopolysaccharide; MUC5AC, mucin 5AC; MTT, 3-(4,5-dimethylthiazol-2yl-)-2,5-diphenyl tetrazolium bromide; HGF, hepatocyte growth factor; SH capsule, Suhuang antitussive capsule.
