# Supplementary material for: Suhuang Antitussive Capsules-Ameliorative Effects on LPS-Induced Sputum Obstruction in Mice Through Promoting HGF Secretion
Source: Front Pharmacol. 2019 Dec 19;10:1422. doi: 10.3389/fphar.2019.01422 (PMC6930918; doi:10.3389/fphar.2019.01422)
Supplement: Supplementary file 1 [file DataSheet_1.docx]

Supplementary Material

**Description of** **SH Capsule is composed of nine traditional Chinese herbs (Species names)**, *i.e.*, *Ephedra sinica* Stapf (Mahuang)*,* *Perilla frutescens* (L.) Britt. (Zisuye), *Pheretima aspergillum* (Dilong), *Cryptotympana pustulata* Fabriciu (Chantui), *Arctium lappa* L. (Niubangzi), *Schisandra chinensis* (Turcz.) Baill. (Wuweizi), *Peucedanum praeruptorum* Dunn (Qianhu), *Eriobotrya japonica* (Thunb.) Lindl. (Pipaye), and *Perilla frutescens* (L.) Britt. (Zisuzi). These original plant specimens were authenticated by Professor Ninghua Tan, Department of TCMs Pharmaceuticals, China Pharmaceutical University, Nanjing, China. The voucher specimens were deposited in Professor Ninghua Tan’s laboratory of China Pharmaceutical University.

**Supplementary Table 1.**

| Formulation | Source | Species | Quality control reported? (Y/N） | Chemical analysis reported? (Y/N) |
| --- | --- | --- | --- | --- |
| SH Capsule | Commercial Supplier, Yangtze River Pharmaceutical Group Beijing Haiyan Pharmaceutical Co., Ltd., Beijing | *Ephedrae Herba*, 0.572 g; *Perillae Folium*, 0.585 g; *Pheretima*, 0.63 g;  *Cicadae Periostracum*, 0.228 g; *Arctii Fructus*, 0.316 g; *Schisandrae Chinensis Fructus*, 0.242 g;  *Peucedani Radix*, 0.231 g; *Eriobotryae Folium*, 0.672 g; *Perillae Fructus*, 0.317 g. | Y - Prepared according to the enterprise quality standard of Yangtze River Pharmaceutical Group Beijing Haiyan Pharmaceutical Co., Ltd., China. | Y - LC-MS |

The extraction of one capsule contains the species section.

**Supplementary Table 2. Primer sequences used in Q-PCR**

| **Gene** | **Forward primer (5’-3’)** | **Reverse primer (5’-3’)** |
| --- | --- | --- |
| Mouse *IL-6* | GCTCTGGCTTGTTCCTCACTACTC | AATGAGGAGACTTGCCTGGTG |
| Mouse *IL-13* | GCTTCGCTTGGTGGTCTCGCC | GGGCTACACAGAACCCCGCA |
| Mouse *MCP-1* | AGGTCCCTGTCATGCTTCTGG | TGCTGCTGGTGATCCTCTTGT |
| Mouse *KC* | GCTGGGATTCACCTCAAGAA | TGGGGACAAATTTTAGCATC |
| Mouse *MUC5AC* | ACATTTCCCCATGCTCCACAGC | GTGGTGGTATTAGACTCCTGG |
| Mouse *HGF* | AGCACCATCAAGGCAAGGT | GACCAGGAACAATGACACCA |
| Human *HGF* | CTCTGGTTCCCCTTCAATAG | GATAGCCCCATTTCTGGATGTC |
| Mouse *GAPDH* | GACATTTGAGAAGGGCCACAT | CAAAGAGGTCCAAAACAATCG |
| Human *GAPDH* | GAAGGTGAAGGTCGGAGTC | GAAGATGGTGATGGGATTTC |

**Supplementary Figure 1**

**A**

Arctigenin

Schisandrin

Arctiin

Ephedrine

Schisandrin B

Schisandrol B

**B**

Ephedrine

Arctiin

Arctigenin

Schisandrin

Schisandrol B

Schisandrin B

**Supplementary Figure 1. LC-MS of SH Capsule.** (**A**) LC total ion chromatogram of SH Capsule and (**B**) MS of the effective components in SH Capsule, i.e., ephedrine, arctiin, arctigenin, schisandrin, schisandrol B, and schisandrin B in SH Capsule were shown.
